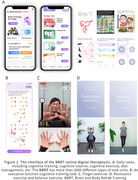# The Role of Emotional Support in Enhancing Adherence to Online Multi‐Domain Cognitive Interventions

**DOI:** 10.1002/alz70860_098957

**Published:** 2025-12-23

**Authors:** Xi Ma, Zhixing Zhou, Chanchan He, Nan Jiang, Huanhuan Xia

**Affiliations:** ^1^ Tsinghua University School of Healthcare Management, Beijing, China; ^2^ Shanghai Bestcovered Limited, Shanghai, China; ^3^ Tsinghua University School of Healthcare Management, Beijing, Beijing, China

## Abstract

**Background:**

Multi‐domain non‐pharmacological interventions, such as cognitive training and physical exercise, have been widely recognized for their effectiveness in improving cognitive function in older adults. However, adherence to such programs, particularly online programs, remains low. This study examines the role of different types of emotional support in relation to adherence to such interventions.

**Method:**

A quasi‐experimental design recruited 524 participants (mean age: 68.4 years, 94.1% female) join in the Brain and Body Rehab Training (BBRT) program (Figure 1), a multi‐domain non‐pharmacological online intervention, for 240 days. Participants were divided into high‐adherence (≥70% task completion, *n* = 250), low‐adherence (30%–70% task completion, *n* = 145), and control (<30% task completion, *n* = 129) groups. Emotional support metrics included community‐based group chat, topic discussion, live classes, one‐on‐one trainer interactions etc. Cognitive performance was measured using the G3 (a three‐minute gamified cognitive screen tool) at baseline, 4 months, and 8 months. Repeated measures analysis evaluated G3 score changes over time, while the Kruskal‐Wallis test compared group differences. Binary logistic regression examined the relationship between emotional support metrics and adherence levels.

**Result:**

Baseline G3 scores did not differ significantly across groups (*p* > 0.05). At 4 months, all groups showed significant G3 score improvements compared to baseline (*p* < 0.05). By 8 months, both the high‐adherence (*p* = 0.029) and low‐adherence (*p* = 0.035) groups exhibited significantly greater G3 score improvements than the control group. Emotional support was significantly associated with adherence. Sharing of check‐in status on the social media (OR = 6.431, *p* = 0.004), participating in community topic discussions (OR = 1.231, *p* = 0.035) and live classes attended (OR = 1.197, *p* = 0.007) were positively associated with adherence, while number of topics shared (OR = 0.393, *p* = 0.015) was negatively associated with adherence.

**Conclusion:**

Emotional support is significantly associated with adherence to online multi‐domain non‐pharmacological interventions among older adults. Tailored strategies, such as live classes and community engagement, are crucial for completing cognitive training and improving cognitive outcomes. Further research is needed to refine these strategies for diverse populations.